# Tumour Volume Independently Predicts Metastasis in ISUP 2 Prostate Cancer

**DOI:** 10.3390/cancers18162605

**Published:** 2026-08-13

**Authors:** David Homewood, Brendan Yanada, Clancy Mullholland, Benjamin Lyttwin, Prabhpreet Mangat, Tanita Botha, Niranjan Sathianathen, George N. Thalmann, Niall M. Corcoran, Marc A. Furrer

**Affiliations:** 1 Department of Urology, Western Health, Melbourne 3011, Australia; davidhom13@gmail.com (D.H.); brendan.yanada@gmail.com (B.Y.); cjpmulholland@gmail.com (C.M.);; 2 Department of Surgery, University of Melbourne, Melbourne 3010, Australia; 3 Department of Urology, University of Bern, 3010 Bern, Switzerland; 4 Department of Urology, Solothurner Spitäler AG, Kantonsspital Olten, 4600 Solothurn, Switzerland; 5 Department of Urology, Solothurner Spitäler AG, Bürgerspital Solothurn, 4500 Solothurn, Switzerland; 6 Biostatistics Unit, Faculty of Health, Deakin University, Geelong 3220, Australia; 7 Department of Urology, Royal Melbourne Hospital, Melbourne 3050, Australia; 8 Department of Urology, Frankston Hospital, Melbourne 3199, Australia; 9 Victorian Comprehensive Cancer Centre, Melbourne 3000, Australia; 10 Department of Anaesthesiology and Pain Medicine, Inselspital, Bern University Hospital, University of Bern, 3010 Bern, Switzerland

**Keywords:** tumour volume, prostate cancer, metastasis-free survival, biochemical recurrence, ISUP 2

## Abstract

**Background and Objectives:** The outcomes for patients with localised prostate cancer are variable, even within defined tumour grades or clinical risk groups. Identifying features associated with disease aggression is imperative in contemporary PCa research. ISUP 2 PCa is particularly important in this regard as it exhibits a wide range of oncologic behaviour. The aim of this study was to evaluate the relationship between the tumour volume at radical prostatectomy (RP) and metastasis-free survival (MFS) in patients with ISUP 2 PCa. **Methods:** In this longitudinal multicentre cohort study, we reviewed the data of 809 consecutive patients with ISUP 2 PCa who underwent RP between 1996 and 2021. The tumour volume was estimated by summing the visual percentage involvement and multiplying it by specimen weight. Kaplan–Meier and Cox regression models were used to assess MFS. Linear regression was used to identify the preoperative predictors of the tumour volume (TV), with the appropriate assumption checks. **Key Findings:** The median TV was 2.9 cc (IQR: 1.4–7.0 cc). The TV categories were <2 cc (37%), 2–20 cc (53%), and >20 cc (11%). The median PSA follow-up was 42 months; 11% experienced biochemical recurrence (BCR). The BCR rates differed significantly across volume categories (*p* < 0.01). At 120 months, MFS was highest in the <2 cc group (97%) and lowest in the >20 cc group (87%) (*p* < 0.01). **Conclusions and Clinical Implications:** Prostate-TV in ISUP-grade group 2 disease is independently associated with MFS. Tumours > 20 cc warrant closer monitoring and may benefit from adjuvant therapy. The improved pre-operative prediction of TV could support more tailored treatment strategies, including suitability for surveillance. **What does the study add?** We demonstrate for first time that tumour volume at prostatectomy is correlated with metastasis-free survival in ISUP 2 PCa. At 120 months, MFS was highest in the <2 cc group (97%) and lowest in the >20 cc group (87%) (*p* < 0.01). This has implications for post-operative surveillance.

## Simple Summary

This study investigated whether tumour volume can predict the risk of cancer spread in patients with ISUP grade group 2 prostate cancer. Researchers reviewed 809 patients who underwent radical prostatectomy between 1996 and 2021. The median tumour volume was 2.9 cc, and patients were divided into three groups: less than 2 cc, 2–20 cc, and more than 20 cc. Larger tumour volumes were associated with higher rates of biochemical recurrence and a greater risk of metastasis. At 120 months, metastasis-free survival was 97% for patients with tumours smaller than 2 cc, compared with 87% for those with tumours larger than 20 cc. Tumour volume above 20 cc remained independently associated with metastasis after adjustment for PSA, pathological tumour stage, and surgical margins. The findings suggest that tumour volume may provide useful additional information for assessing prognosis and treatment decisions in ISUP 2 prostate cancer. However, the study’s retrospective design, low number of metastatic events, and measurement limitations mean that further prospective research is needed.

## 1. Introduction

There is significant biological heterogeneity in prostate cancer (PCa), which contributes to variations in aggressiveness, the risk of metastasis, and, ultimately, patient prognosis. Whilst the pathological grade successfully stratifies this to some extent, finding biomarkers, genetic factors, and radiological and pathological features to more accurately predict the natural history is a key focus of contemporary PCa research [1]. ISUP grade group 2 PCa is particularly important in this regard, as it is the most commonly diagnosed tumour type and exhibits a large variation in oncologic behaviour [2]. Effectively stratifying the metastatic risk at the time of diagnosis enables the development of tiered treatment protocols with prognosis- and risk-appropriate therapies. The tumour volume (TV) has been proposed as a tool to do this, but its independent prognostic value is not proven [3].

The TV in PCa specimens following RP can be estimated using several methods, each varying in accuracy and complexity [4]. The ellipsoid method employs a mathematical formula to calculate the volume based on the largest tumour dimensions and a correction coefficient [5]. Sectional measurement, a more precise but time-intensive technique, sums the tumour areas across all prostate sections. Advanced computer-aided 3D reconstruction uses pathological data to create detailed models for precise volume estimation [6].

Whilst such sophisticated methods generally yield more precise and accurate results, they are not pragmatic in clinical settings [7]. Visual estimation for the tumour volume percentage, though less accurate, is more practical and widely used for the histopathological examination of prostatectomy specimens [7]. The TV is a calculated variable through multiplying an estimation of the tumour percentage involvement/100 by prostate volume (through weight-to-volume conversion). This gives a TV in cubic centimetres (cc).

The TV has been demonstrated to independently predict biochemical recurrence (BCR) in PCa, but its role in predicting metastasis is less clear [8,9,10,11]. Studies highlight several key findings: the maximum tumour diameter significantly predicts BCR [8]; the TV assessed post-surgery is an independent risk factor for BCR [11]; and the tumour–prostate ratio may outperform the TV alone in predicting BCR [11]. Additionally, semiautomatically quantified TV using 68Ga-PSMA-11 PET/CT could predict the overall survival in advanced PCa [12], and the TV percentage (TVP) with a cut-point of ≥8% offers robust discrimination for BCR post-RP [13]. However, post-prostatectomy BCR is a poor surrogate of MFS and prostate-cancer-specific survival. In addition, multivariate regression models to predict metastasis require consideration of other factors such as the Gleason score, pathological stage, and surgical margin status [14].

Established TV cutoffs of 2.8 cc have been postulated as an independent predictor of BCR [9]. However, whilst the TV correlated with the grade and stage and there is some evidence to demonstrate an effect on BCR [15], there is a paucity of data to show that the TV independently predicts metastasis-free survival [16,17].

Robust data on the effect of TV in ISUP 2 PCa on metastasis-free survival remains sparse.

The aim of this study was to evaluate the relationship between the TV at RP and MFS in patients with ISUP 2 PCa.

## 2. Materials and Methods

### 2.1. Patient Population

In this longitudinal multicentre cohort-study, we reviewed data of 809 consecutive patients with ISUP 2 PCa who underwent RP at a total of 5 institutions, including the University Hospital of Bern, The Royal Melbourne Hospital, Epworth Health Care, Peter MacCallum Cancer Centre, and St. Vincent’s Hospital between January 1996 and December 2021. The cohort was >95% of European ancestry, and all patients were assessed as having a life expectancy >10 years at diagnosis.

The study was conducted in accordance with the Strengthening the Reporting of Observational Studies in Epidemiology (STROBE) statement and approved by the Ethics Committee of Canton Bern, Switzerland (KEKBE 2016-00156) and Melbourne Health (QA2020180).

### 2.2. TV Calculation and Categorisation

TV was determined post-operatively using visual estimates of tumour percentage/100 provided by pathologists, multiplied by specimen weight. Tumour volume estimation was based on visual assessment without formal standardisation across institutions. TV was categorised into three ordinal groups—<2 cc, 2–20 cc, and >20 cc—based on pragmatic thresholds informed by prior literature but not biologically defined, reflecting low-, intermediate-, and high-volume diseasem respectively, to facilitate risk stratification and align with established prognostic cut-offs. These empirically derived thresholds allowed for clinically meaningful stratification in evaluating prostate cancer outcomes.

### 2.3. Oncological Outcomes

PSM was defined as malignant cells at the inked margin of the prostatectomy specimen. BCR was defined as PSA ≥ 0.2 ng/mL in two consecutive measurements [18]. With a rising PSA and/or clinical suspicion, imaging (bone scan and/or CT and/or PET-CT) was obtained to assess the amount and localization of tumour recurrence. We registered and counted separately any metastases, local recurrence, bone metastases, and lymph metastases.

We used an established broad definition of metastasis to include recurrence in pelvic regional lymph nodes in addition to lymph node metastases outside of the pelvis, as well as bony and visceral metastasis [19]. This composite endpoint was selected to maximise event capture but may obscure differences between regional and distant disease. Metastases were identified by conventional imaging post-prostatectomy.

### 2.4. Surgical Procedure

A standardized surgical technique was performed either via an open retropubic or robot-assisted laparoscopic approach.

### 2.5. Statistical Analysis

Categorical variables are presented as number (percentage), while continuous variables were reported using the mean (SD) or median (IQR) where appropriate. The normality of data was assessed using the Shapiro–Wilk test, while correlation investigations included Spearman’s correlation, since the normality assumption did not hold. Kaplan–Meier survivorship curves and Cox hazard regression was used to perform survival analysis. Linear regression was performed to assess the relationship between tumour weight and clinical predictors, weighted by MRI TV. Statistical significance was set at *p* < 0.05, and analyses were conducted using R (version 4.4.2) [20].

## 3. Results

A total of 809 ISUP 2 patients were included for retrospective analysis. The clinical and pathological characteristics of the cohort are outlined in Table 1. The median age was 61 (IQR: 57–66). The median PSA was 6.07 (IQR 4.68–8.40). Regarding the pathological stage, 606 (74.9%) were pT1–pT2, 180 (22.2%) were pT3a, and 23 (2.8%) were pT3b–pT4. 620 (76.6%) patients had negative surgical margins, whilst 189 (23.4%) had positive surgical margins. The median prostate volume was 46 cc (IQR: 37 cc–57 cc). The median TV was 2.90 cc (IQR: 1.40 cc, 7.00 cc). As outlined in the methods, patients’ TV was split into ordinal categories, with 296 (36.6%) less than 2 cc, 426 (52.7%) 2 to 20 cc, and 87 (10.8%) greater than 20 cc.

### 3.1. TV Independently Predicts BCR

**Figure 1:**
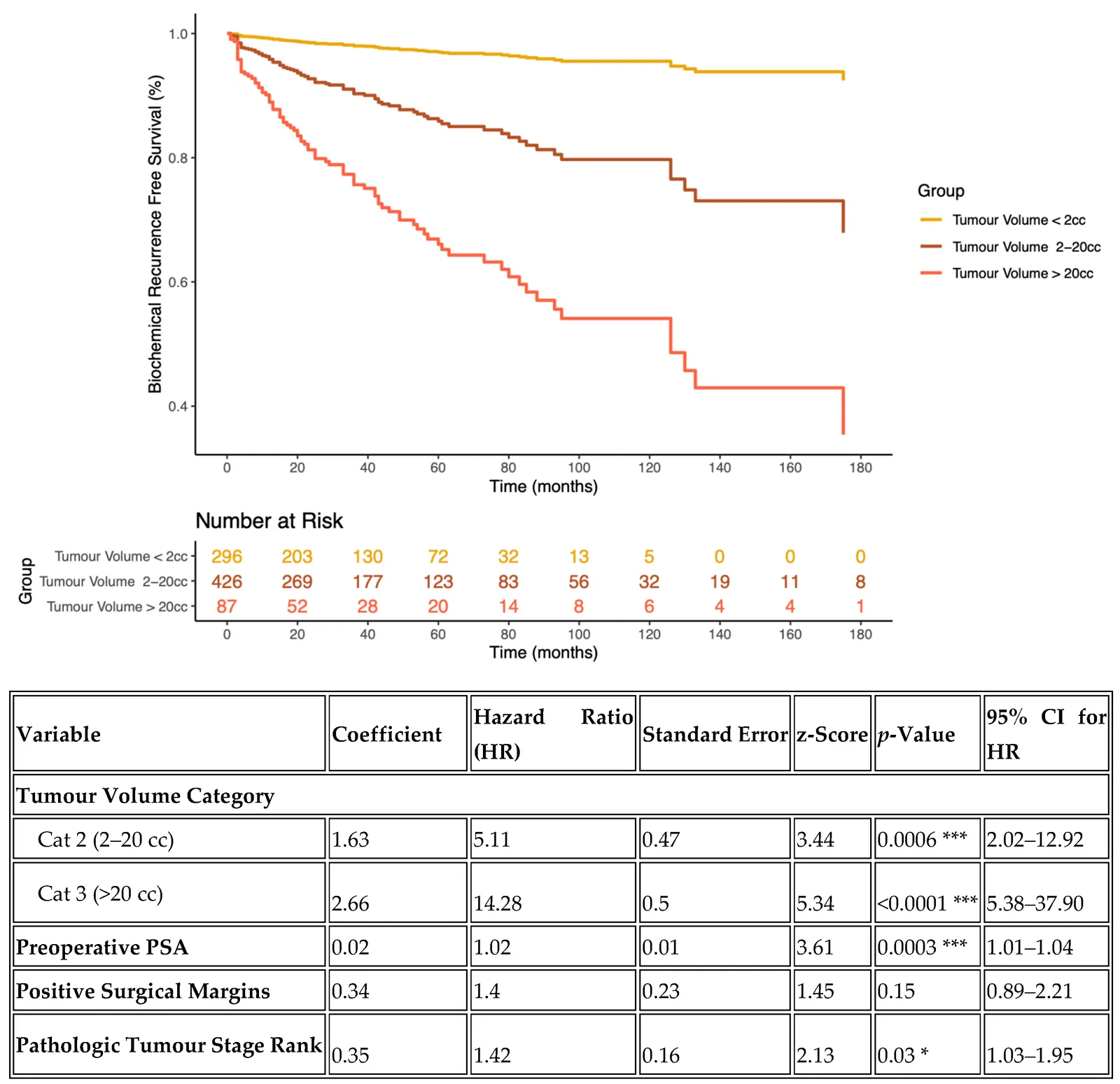
Tumour volume independently predicts BCR (adjusted for pre-operative PSA, positive surgical margins, and pT stage). Significance codes: <0.001 ***|<0.05 *.

The mean PSA follow-up duration was 42 months (SD 39.5). BCR occurred in 90 patients (11%), while 719 (89%) maintained a PSA <0.2 ng/mL. When stratified by TV, BCR rates were 1.7% for <2 cc, 11.3% for 2–20 cc, and 42.5% for >20 cc, with median times to BCR of >180, ~112, and ~58 months, respectively. The Kaplan–Meier analysis adjusted for PSM, PSA and pT rank demonstrated significant differences in BCR-free survival between <2 cc vs. 2–20 cc and 2–20 cc vs. >20 cc (Figure 1). Using multivariable Cox regression, it was found that an increasing TV was independently associated with a greater risk of BCR: 2–20 cc HR 5.1 (95% CI 2.0–12.9) and >20 cc HR 14.2 (95% CI 5.4–37.9).

### 3.2. TV Independently Predicts MFS

Over a mean follow-up period of 47 months, 24 patients met the study definition of metastatic disease. A multivariate analysis reveals that tumours measuring 2–20 cc (Category 2) showed a trend toward increased risk (HR 7.63, 95% CI 0.99–58.62), which did not reach statistical significance (*p* = 0.051), whereas those exceeding 20 cc (Category 3) were associated with a higher hazard ratio (HR 14.75), although the estimates were imprecise (95% CI 1.68–129.35) (*p* = 0.015). The model was adjusted for preoperative PSA (HR: 1.03, *p* = 0.042), pathologic tumour stage rank (HR: 2.30, *p* = 0.006), and positive surgical margins (*p* = 0.375).

### 3.3. TV of >20 cc Significantly Increases Risk of Metastasis

**Figure 2:**
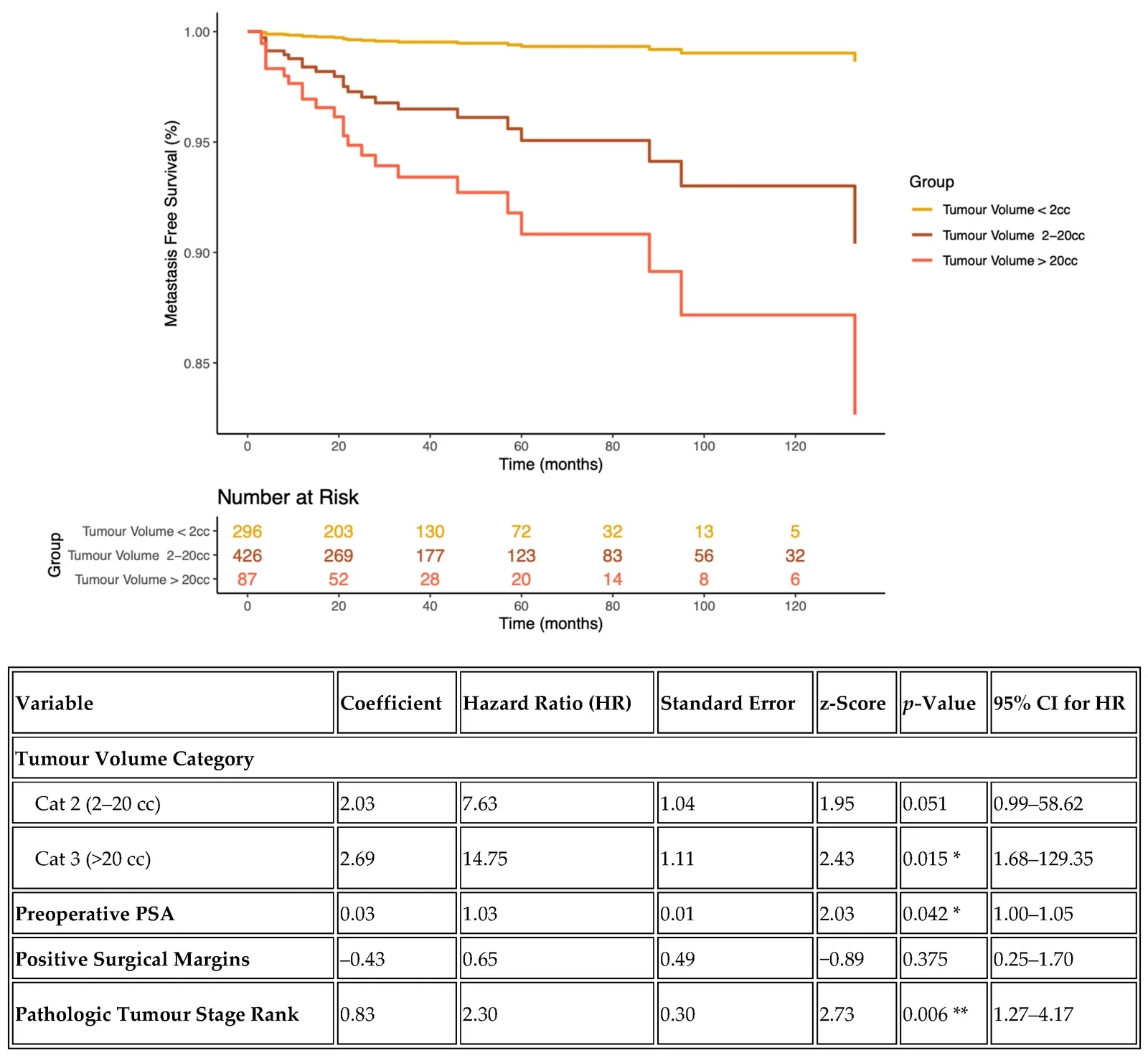
Tumour volume independently predicts metastasis in ISUP 2 PCa (adjusted for pre-operative PSA, positive surgical margins, and pT stage). Significance codes: <0.01 **|<0.05 *.

A further analysis using Kaplan–Meier survival curves demonstrated a significant difference in metastasis-free survival (MFS) across TV groups adjusted for positive surgical margins, PSA, and pT stage. At 120 months, MFS was highest in patients with TV < 2 cc (97%) and lowest in those with volume > 20 cc (87%) (*p* < 0.001) (Figure 2). On a multivariable Cox regression analysis, TV >20 cc was independently associated with an increased risk of metastasis (HR 14.75; 95% CI 1.68–129.35; *p* = 0.015), while TV 2–20 cc had no association (HR 7.63; 95% CI 0.99–58.62; *p* = 0.051). Other independent predictors of metastasis included preoperative PSA (HR 1.03 per unit increase; *p* = 0.042) and a higher pathological tumour stage (HR 2.30; *p* = 0.006).

### 3.4. TV of 2 cc Trends Towards a Tipping Point for Hazard of Metastasis

**Figure 3:**
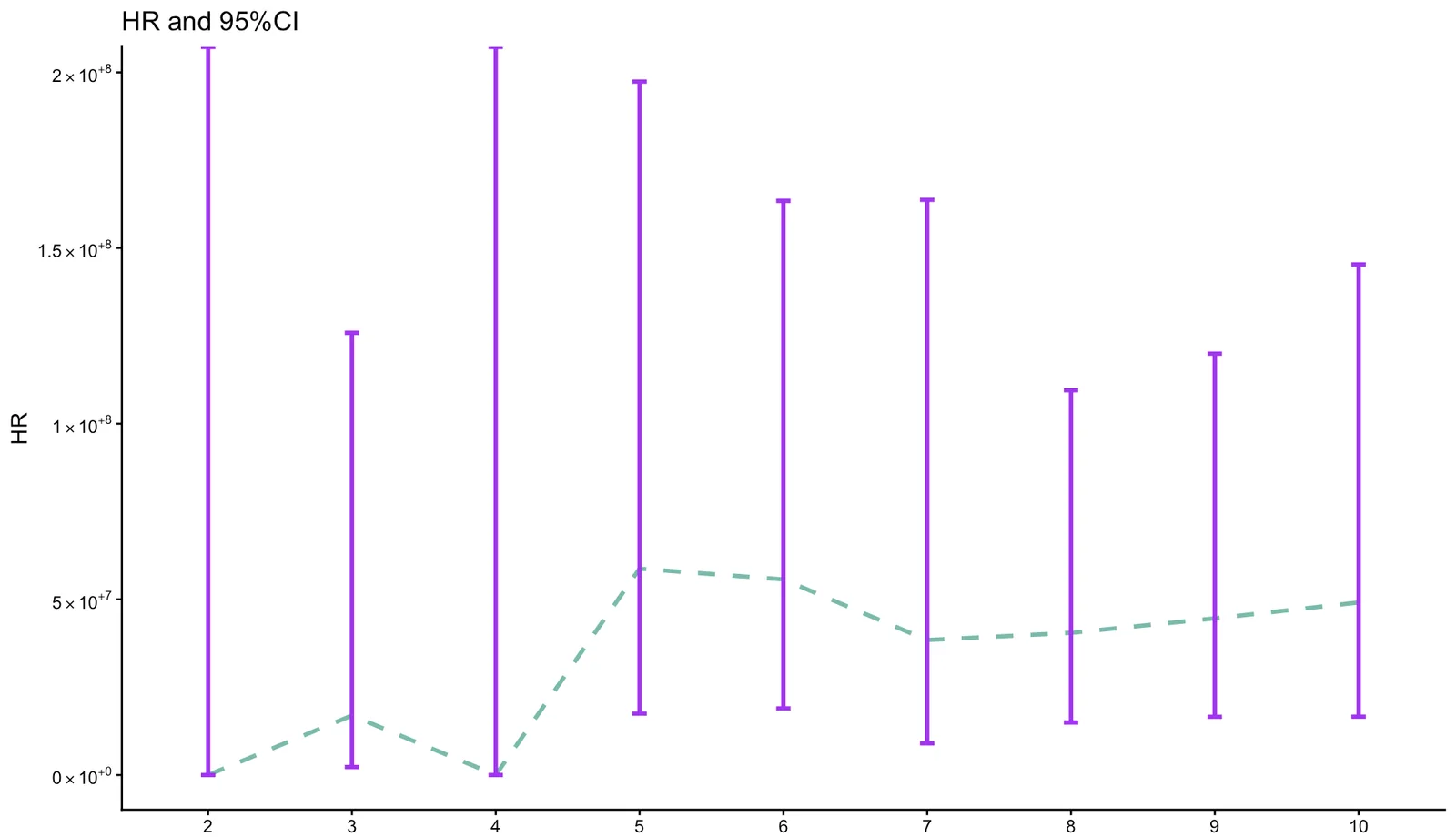
Association between tumour volume and hazard of metastasis, displayed by decile. This graph shows the estimated hazard ratio (HR) for metastasis (y-axis) across deciles of tumour volume (x-axis), with 95% confidence intervals. The dashed line represents the estimated HR across tumour-volume deciles, and the vertical error bars represent the corresponding 95% confidence intervals. Tumour volume was divided into ten equal-sized groups (deciles), and the hazard ratio for each decile was computed relative to the lowest-volume group. The wide confidence intervals in higher deciles indicate statistical uncertainty due to fewer events or data variability. A sharp increase in hazard ratio occurs between the 4th and 5th deciles—corresponding to a tumour volume of approximately 2 cm^3^—suggesting a potential threshold beyond which the risk of metastasis substantially increases.

The TV was split into deciles with the associated hazard ratio of metastasis calculated (Figure 3). This demonstrated a trend of an increased hazard of metastasis in between the fourth and fifth decile which correlated with approximately 2 cc (note that this tipping point indicates the transition from a no–low chance of metastatic disease to an unsure risk of metastatic disease given the power limitations). Given the wide confidence intervals and limited event numbers, this observation should not be interpreted as a definitive biological threshold.

## 4. Discussion

Our findings suggest an association between an increasing tumour volume and adverse oncological outcomes in ISUP grade group 2 prostate cancer. This observation is consistent with established oncological principles across solid tumours and should be interpreted as confirmatory within this clinically heterogeneous subgroup rather than representing a novel biological finding. In this context, the TV independently predicts both BCR and MFS in ISUP grade group 2 disease. Furthermore, the TV of >20 cc significantly increases the risk of metastasis while the TV of >2 cc may suggest a potential transition point for the significant increase in the risk of post-operative metastatic risk.

### 4.1. Clinical Significance of TV

As aforementioned, the TV has been demonstrated to independently predict BCR in PCa, but its role in predicting metastasis has thus far been less clear [9,11]. Our findings highlight the clinical significance of the TV, grouped into ordinal categories in PCa prognosis, specifically MFS. Our findings demonstrate a progressive increase in risk with rising TVs, reinforcing the value of the TV as a prognostic factor along a continuum. However, statistically and clinically significant thresholds appeared to emerge at approximately 2 cc and 20 cc, indicating the categorisation of the TV into low-volume (<2 cc), intermediate-volume (2–20 cc), and high-volume (>20 cc) provides practical, clinically meaningful distinctions.

### 4.2. Implications for Selection of Patients for AS

Current AS guidelines for prostate cancer primarily select patients based on clinical stage, PSA, PSA density, number of positive biopsy cores, and ISUP grade group, with ISUP 2 patients often included only if other risk factors are favourable [3]. As our data show that tumour volume is an independent predictor of metastasis in ISUP 2 cancers, its formal inclusion in AS eligibility criteria warrants consideration. Traditional triggers for treatment escalation included increasing PSA, and upstaged or upgraded disease. These findings suggest that estimating the tumour volume from MRI and biopsy data may allow for a more precise clinical risk stratification.

### 4.3. Implications on Adjuvant Hormonal Treatment in Patients with High TV (>20 cc)

The strong correlation between the TV and metastasis-free survival, particularly the significantly increased risk for tumours >20 cc (HR 14.75, *p* = 0.015), suggests the need for studies assaying the utility of treatment intensification in this cohort. Although our data does not directly evaluate such therapies, the notably lower MFS observed in patients with TVs > 20 cc (87% vs. 97% at 120 months for tumours < 2 cc, *p* < 0.001) implies the role of a further study on using aggressive treatment approaches for those with very large tumour burdens, as adjuvant hormonal therapy has shown benefits for patients with high-risk PCa in other studies, improving survival and potentially delaying metastasis [21]. However, while patients with a higher tumour volume demonstrated worse outcomes, this study did not evaluate the impact of adjuvant or salvage therapies. As such, any suggestion that the tumour volume should guide adjuvant hormonal therapy is speculative and not supported by the present data. Prospective studies would be required to determine whether the tumour volume has a role in informing treatment intensification strategies.

### 4.4. Recent Advances in Estimation Techniques of TV

Multiparametric MRI predicts the prostate cancer tumour volume more accurately than serum PSA or the clinical Epstein criteria, which underestimate the clinically significant disease in 24% of cases [22]. Although MRI can delineate the shape and size of most clinically significant PCa, it can underestimate TV and may miss other clinically significant tumours and satellite lesions [23]. Various methods have been proposed for quantifying the TV on MRI such as using maximal tumour diameter, ellipsoid formula, or planimetry; however, there remains a lack of consensus regarding the method of measuring the TV on multiparametric MRI [24]. The more novel PSMA PET CT imaging modality has demonstrated modest improvements over MRI in predicting the TV despite its lower tissue resolution and soft-tissue contrast [25].

Techniques that incorporate imaging with additional parameters such as the positive core length on targeted prostate biopsy have been described and shown to increase the accuracy of the predicted TV compared with MRI alone and allow for better risk stratification [26]. Emerging protocols using a multimodal approach by combining the TV with genomic classifiers and MRI characteristics may enhance the risk stratification of BCR and metastatic disease and adverse pathological features [27]. It is predicted that artificial intelligence (AI) may be used to incorporate multimodal information from imaging, PSA, and prostate biopsy, and will continue to enhance the precision of the estimated TV and margins, optimising treatment decision-making and surgical planning, particularly in the novel field of ablative focal therapy [28].

### 4.5. Other Implications on Clinical Practice

ISUP 2 (Gleason score 3 + 4 = 7) PCa remains highly controversial and multifaceted with a wide variety of management options ranging from active surveillance to curative therapies including RP, radiation therapy, and ablative therapies, and, more recently, novel focal therapy [29,30].

Various focal therapies such as high-intensity focused ultrasound (HIFU), cryotherapy, focal laser ablation, focal brachytherapy, radiofrequency ablation (RFA), photodynamic therapy, and interstitial drug injections have been developed to minimise treatment toxicity while selectively ablating prostate cancer [24]. Precise TV estimation is crucial in focal therapy for accurate cancer mapping, facilitating the optimal energy delivery and minimizing the residual disease to improve outcomes such as BCR and MFS [24]. Despite the evidence in favour of focal therapy in patients with localised PCa and the importance of an accurate assessment of the TV and careful patient selection prior to embarking on this form of therapy, the reported studies exhibit considerable variability in terms of the patient selection criteria and treatment planning approaches [31].

Patients with larger TVs may require more aggressive treatments such as RP or external beam radiotherapy to adequately treat the cancer in its entirety [10,31]. High TVs are known to correlate with adverse pathological outcomes including extraprostatic extension and positive surgical margin; hence, an accurate pre-operative assessment has a role in guiding surgical technique (such as the decision to perform a nerve-sparing technique) [31].

### 4.6. Potential Biological Mechanisms Underlying TV and Poor Oncological Outcomes

The association between tumour volume and worse oncologic outcomes is biologically plausible. Larger tumours likely harbour a greater clonal diversity and intratumoral heterogeneity, increasing the chance that invasive or metastatic subclones emerge and are selected for during growth [32]. They may also carry a greater genomic instability and tumour mutational burden, with accumulated driver alterations (e.g., TP53, PTEN, and MYC) contributing to adverse behaviour independent of the ISUP grade [33]. This could potentially explain why the volume retains an independent prognostic value in our models. Larger tumours are also more prone to hypoxic cores, which promote angiogenesis (e.g., HIF-1α/VEGF signalling) and treatment-resistant phenotypes, and may be associated with a more immunosuppressive microenvironment (reduced cytotoxic T-cell infiltration, increased regulatory T cells, or myeloid-derived suppressor cells) that facilitates immune evasion [34]. Finally, an increasing volume raises the likelihood of subclinical extraprostatic extension and micrometastatic seeding at surgery, independent of the grade. While our data cannot test these mechanisms directly, they offer plausible biological explanations for our findings and motivate future work incorporating genomic or microenvironment profiling in ISUP 2 disease.

### 4.7. Limitations

This study has several important limitations. First, the retrospective design and long inclusion period (1996–2021) introduce substantial heterogeneity in diagnostic, surgical, and pathological practices, which may confound results. Second, the tumour volume was estimated visually across multiple institutions without a formal assessment of the interobserver variability, introducing potential measurement bias. This is a significant limitation and future work with robust standardisation processes will be needed to address this bias. Third, the number of metastatic events was low, resulting in wide confidence intervals and potential overfitting in multivariable models; therefore, the estimates should be interpreted with caution. Additionally, key patient-level factors such as comorbidities, detailed age stratification, and genomic data were not available, limiting the adjustment for confounding. There was also limited data collected on the rates of patients subsequently receiving salvage or adjuvant therapies, which has implications for the rates of subsequent rates of metastatic disease. The cohort was also relatively homogeneous in ancestry, which may affect generalisability. Further to this, a temporal sensitivity analysis was not performed due to the low metastasis event count, which may also limit the generalisability of our findings to the current clinical practice given the changes in imaging, biopsy, and surgical technique over the study period.

Given the limited number of metastatic events, multivariable models may be underpowered and prone to overfitting.

Additionally, since most patients underwent surgery prior to the widespread uptake of PSMA-PET/CT pre-operative staging, we cannot comment on its impact on accuracy in pre- and postoperative N- and M-staging. This is due to PSMA-PET with 18F-PSMA only being authorised in Switzerland from October 2019, and the [68Ga] Ga-PSMA-11 radiolabelling kit only being approved for clinical use by the Australian Therapeutic Goods Administration in 2021. In Switzerland, PSMA PET has not been routinely used in preoperative staging. Whilst PSMA-PET/CT shows superior accuracy for staging compared to CT and a bone scan for high-risk disease, no longitudinal outcome data currently exist demonstrating its superiority to inform subsequent management.

To the best of our knowledge, this is the only multicentre study assessing the impact of the TV at RP and MFS in patients with ISUP 2 PCa. The data provided are not only useful for surgeons’ decision-making peri- and postoperatively, including long-term follow-up, but also patient counselling and management. However, further long-term data obtained from prospective trials are needed to make definitive decisions about how the TV affects the suitability for AS or adjuvant hormonal therapies.

## 5. Conclusions

The tumour volume at prostatectomy is associated with adverse oncological outcomes in ISUP grade group 2 prostate cancer; however, given the methodological limitations, low event rates, and imprecision of the estimates, these findings should be considered exploratory. The tumour volume may serve as an adjunct to established prognostic factors, but its clinical utility requires validation in contemporary cohorts incorporating biological, treatment, and imaging data. Further work should explore methods to more accurately preoperatively predict the prostatectomy TV to improve definitive treatment decisions.

## Figures and Tables

**Table 1:** Descriptive data from multi-institutional international registry for ISUP 2 Prostate Cancer.

	*n* = 809
Age (y)	
Median (IQR)	61.00 (57.00, 66.00)
PSA (ng/mL)	
Mean (SD)	7.81 (7.15)
Pathological T categories	
pT1-pT2	606 (74.9%)
pT3a	180 (22.2%)
pT3b-pT4	23 (2.8%)
Positive Surgical Margin	
Yes	189 (23.4%)
No	620 (76.6%)
Prostate Volume (cc)	
Median (IQR)	46.00 (37.00, 57.00)
Tumour Volume (cc)	
Median (IQR)	2.90 (1.40, 7.00)
Tumour Volume Category n (%)	
<2 cc	296 (36.6%)
2–20 cc	426 (52.7%)
>20 cc	87 (10.8%)
BCR n (%)	
Yes	90 (11.1%)
No	719 (88.9%)
BCR Follow-Up Time (months)	
Mean (SD)	42.70 (39.54)
Metastasis n (%)	
Yes	24 (3.0%)
No	785 (97.0%)
Mets Follow-Up Time (months)	
Mean (SD)	47.91 (43.74)

## Data Availability

The raw data supporting the conclusions of this article will be made available by the authors upon request.
